# KLF5 controls glutathione metabolism to suppress p190-BCR-ABL+ B-cell lymphoblastic leukemia

**DOI:** 10.18632/oncotarget.25667

**Published:** 2018-07-03

**Authors:** Cuiping Zhang, Angelo D'Alessandro, Ashley M. Wellendorf, Fatima Mohmoud, Juana Serrano-Lopez, John P. Perentesis, Kakajan Komurov, Gabriela Alexe, Kimberly Stegmaier, Jeffrey A. Whitsett, H. Leighton Grimes, Jose A. Cancelas

**Affiliations:** ^1^ Division of Experimental Hematology and Cancer Biology, Cincinnati Children’s Hospital Medical Center, Cincinnati, OH, USA; ^2^ Department of Biochemistry and Molecular Genetics, University of Colorado Denver-Anschutz, Aurora, CO, USA; ^3^ Hoxworth Blood Center, University of Cincinnati, Cincinnati, OH, USA; ^4^ Department of Oncology, Cincinnati Children’s Hospital Medical Center, Cincinnati, OH, USA; ^5^ Department of Pediatric Oncology, Dana-Farber Cancer Institute and Boston Children's Hospital, Harvard Medical School, Boston, Massachusetts, Boston, MA, USA; ^6^ Broad Institute of Harvard University and Massachusetts Institute of Technology, Cambridge, MA, USA; ^7^ Pulmonary Biology, Cincinnati Children’s Hospital Medical Center, Cincinnati, OH, USA; ^8^ Immunobiology and Center for Systems Immunology, Cincinnati Children’s Hospital Medical Center, Cincinnati, OH, USA

**Keywords:** preB-lymphoblastic leukemia, philadelphia+, KLF5, GSTM1, metabolome

## Abstract

High-risk B-cell acute lymphoblastic leukemia (B-ALL) remains a therapeutic challenge despite advances in the use of tyrosine kinase inhibitors and chimeric-antigen-receptor engineered T cells. Lymphoblastic-leukemia precursors are highly sensitive to oxidative stress. KLF5 is a member of the Krüppel-like family of transcription factors. KLF5 expression is repressed in B-ALL, including BCR-ABL1+ B-ALL. Here, we demonstrate that forced expression of KLF5 in B-ALL cells bypasses the imatinib resistance which is not associated with mutations of BCR-ABL. Expression of Klf5 impaired leukemogenic activity of BCR-ABL1+ B-cell precursors *in vitro* and *in vivo*. The complete genetic loss of Klf5 reduced oxidative stress, increased regeneration of reduced glutathione and decreased apoptosis of leukemic precursors. Klf5 regulation of glutathione levels was mediated by its regulation of glutathione-S-transferase Mu 1 (*Gstm1*), an important regulator of glutathione-mediated detoxification and protein glutathionylation. Expression of Klf5 or the direct Klf5 target gene *Gstm1* inhibited clonogenic activity of *Klf5*^∆/∆^ leukemic B-cell precursors and unveiled a Klf5-dependent regulatory loop in glutamine-dependent glutathione metabolism. In summary, we describe a novel mechanism of Klf5 B-ALL suppressor activity through its direct role on the metabolism of antioxidant glutathione levels, a crucial positive regulator of leukemic precursor survival.

## INTRODUCTION

Philadelphia chromosome-positive (Ph+) hematological malignancies arise from the reciprocal translocation, t(9,22) (q34;q11.2), which encodes the protein BCR-ABL1. BCR-ABL1 is the transformation driver of ∼5–30% of B-ALL in patients [1, 2] where the expression of the p190 form of BCR-ABL1 is the most frequent form in children and is associated with the transformation of an early B-cell precursor. Expression of BCR-ABL1 in B-ALL confers a much poorer prognosis compared to other cytogenetic or molecular abnormalities (reviewed in [3]).

The ABL tyrosine kinase inhibitors (TKI) are only partly effective in Ph+ B-ALL patients. The long-term survival rate is about 80% for children in their first complete remission. Clinical outcome for adult ALL is worse, with few survivors at 10 years. The failure of TKI therapy is due to resistance-inducing mutations and primary resistance of leukemic progenitors/precursors to TKI therapy [4]. While resistance-inducing mutations have been successfully targeted with novel inhibitors like dasatinib, nilotinib or ponatinib (reviewed in [5]), acquired resistance due to genomic events resulting in activation of alternative signaling pathways and non-oncogene addiction (reviewed in [6]) contribute to the poor prognosis of these patients, whose only hope frequently relies on chimeric antigen receptor transgenic T-cell (CAR-T) therapies [5]. Identification and targeting of downstream signaling cascades by BCR-ABL1 may lead to more effective primary therapeutic strategies with the aim of preventing the development and/or selection of leukemic clones that are resistant to TK inhibition. Multi-targeted approaches are therefore appropriate in treating Ph+ leukemia.

Multiple signaling mechanisms are induced by BCR-ABL1. The role of BCR-ABL1 as an activator of reactive oxygen species (ROS) that drive transformation is well established [7]. Paradoxically, treatment with antioxidants increases proliferation of BCR-ABL1 leukemic cells [8].

Glutathione-S-transferases (GSTs) are a superfamily of dimeric phase II detoxification enzymes that catalyze the conjugation of reduced glutathione (GSH) to various endogenous and exogenous toxic and carcinogenic electrophilic compounds, and play an important role in cellular protection from environmental and oxidative stress. Mammalian cytosolic GSTs are subdivided into seven distinct classes designated as: α (A), μ (M), π (P), σ (S), θ (T), ω (O) and ζ (Z) [9]. Recent studies demonstrated the role of GST polymorphisms in cancer susceptibility. GSTs are important in the modulation of the biological effects of carcinogens and the metabolism of a broad range of ROS and xenobiotics [9]. The *GSTM1*-null genotype was found to be associated with an increased risk for childhood ALL. The *GSTT1*-null genotype was associated with an increased risk for ALL, primarily when combined with the dominant *GSTM1*-null genotype [10].

KLF5 belongs to the Krüppel-like factor (KLF) family of zinc-finger-containing transcription factors, which regulate diverse functions, including proliferation, self-renewal, survival, differentiation and development [11, 12]. Klf5 generally acts as a transcriptional activator that is dysregulated in many cancers. Functional studies support *KLF5* as an important cancer-related gene.

In intestine, colon, breast, bladder and pancreatic cancers, KLF5 enhances cell proliferation, survival and invasiveness, and is considered to act as an oncogene. However, in prostate, esophageal squamous cell cancers and acute myeloid leukemia (AML), KLF5 inhibits cell proliferation and promotes cell differentiation, acting as a tumor suppressor (reviewed in [13]). Recent data supports the function of *KLF5* as an oncogene or tumor suppressor in carcinogenesis depending on the cellular and genetic context in which it operates [11].

KLF5 is an unstable protein with a short half-life [14] and multiple mechanisms of ubiquitination/deubiquitination have been implicated in its expression [15–17]. In some types of B-ALL, KLF5 has been found to function as an oncoprotein in complex with p53 to regulate survivin transcriptional activity [18]. However, the *KLF5* promoter has been found to be hyper-methylated in BCR-ABL1 expressing B-ALL [19], suggesting that KLF5 transcriptional regulation may be relevant and as such it may act as a tumor suppressor in this specific type of leukemia. In this report, we identify the role of KLF5 as a suppressor of BCR-ABL1 B-ALL, and compared its activity in Ph+ B-ALL and non-Ph+ B-ALL.

## RESULTS

### KLF5 level is decreased in BCR-ABL1+ B-ALL leukemia

**Figure 1:**
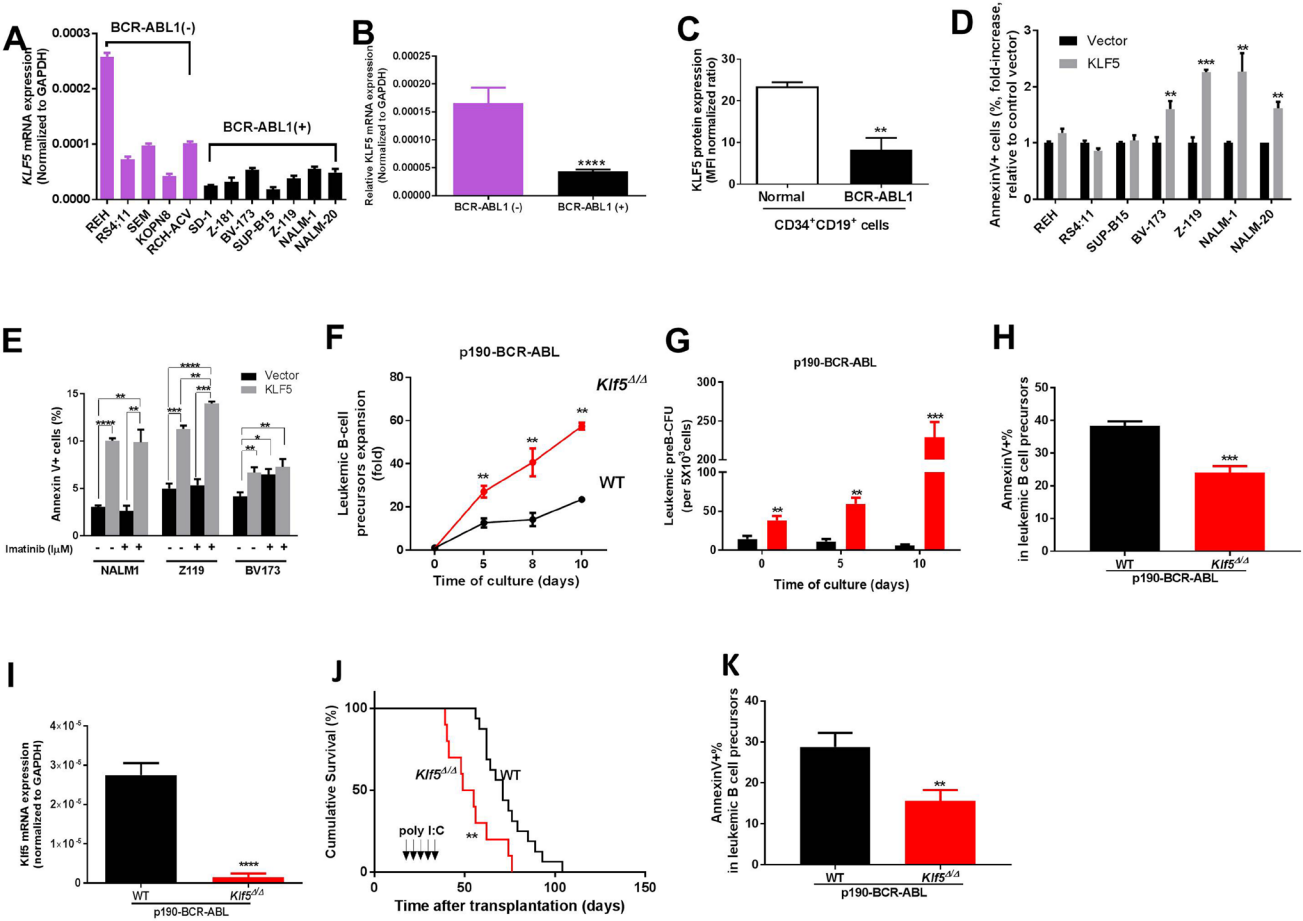
Klf5 is a tumor suppressor of BCR-ABL transformed leukemogenesis through promotion of apoptosis of B precursor cells (**A**) *KLF5* mRNA expression in human B-ALL cell lines grouped according to their BCR-ABL expression (BCR-ABL-negative lines in purple; BCR-ABL positive lines in black). Two independent experiments were performed in triplicate from the same samples and the data are given as mean ± SEM. (**B**) The difference of *KLF5* mRNA expression in human B-ALL cell lines between BCR-ABL-negative and BCR-ABL-positive group (from Figure 1A). (**C**) Flow cytometry analysis of KLF5 protein expression in normal CD34+CD19+ BM cells (empty bar, *n =* 3) and BCR-ABL1+ CD34+CD19+ BM from B-ALL patients CD34+CD19+cells (black solid bar, *n =* 3). Values represented as mean ± SEM. (**D**) Apoptosis as assessed by fold increase in annexin V+ cell percentage of B-ALL cell lines transduced with either KLF5 (grey solid bars) or empty (black solid bars) vectors. Data derived from two independent experiments. Each experiment was performed in duplicate and data are given as mean ± SEM. (**E**) Apoptosis as assessed by annexin V+ cell percentage in NALM-1, Z-119 and BV-173 cells transduced with KLF5 (grey bar) or empty (black bar) vectors in 24-hour cultures with or without imatinib (1 mM). (**F**) Expansion (fold) of *in vitro*-cultured B-cell precursors from *Vav1-Cre*; WT (black line and symbols) or *Vav1-Cre; Klf5*^*flox/flox*^ (red line and symbols) mice transduced with p190-BCR-ABL. Data derived from two independent experiments. Each experiment was performed in triplicate and data are given as mean ± SEM. (**G**) CFU-preB colony formation (*in vitro* culture at Days 0, 5, and 10 after sorting) of sorted p190-BCR-ABL transduced B-cell precursors from *Vav1-Cre*; WT (WT, black solid bars) or *Vav1-Cre; Klf5*^*flox/flox*^ mice (*Klf5*^Δ/Δ^, red solid bars). Data derived from two independent experiments. Each experiment was performed in triplicate and data are given as mean ± SEM. (**H**) Apoptosis assessed by annexin V+ cell percentage of p190-BCR-ABL transduced, *in vitro* generated B-cell precursors from *Vav1-Cre*; WT (WT, black solid bars) or *Vav1-Cre*; *Klf5*^*flox/flox*^ mice (*Klf5*^Δ/Δ^, red solid bars). Two independent experiments were performed in triplicate and data are given as mean ± SEM. (**I**) *Klf5* mRNA expression of p190-BCR-ABL leukemic B-cell precursors from *Mx1-Cre*; WT (WT, black solid bars, *n =* 7) or *Mx1-Cre; Klf5*^*flox/flox*^ (*Klf5*^Δ/Δ^, red solid bars, *n =* 9) mice. Values are given as mean ± SD. (**J**) Survival of mice transplanted with 1 × 10^6^ p190-BCR-ABL transduced LDBM cells from *Mx1-Cre*; WT (WT, black line and symbols, *n =* 16), or *Mx1-Cre; Klf5*^*flox/flox*^ *(Klf5*^Δ/Δ^, red line and symbols, *n =* 10) mice. (**K**) Apoptosis assessed by annexin V+ cell percentage of p190-BCR-ABL + B-cell precursors from *Mx1-Cre*; WT (WT, black solid bars, *n =* 4) or *Mx1-Cre; Klf5*^*flox/flox*^ (*Klf5*^Δ/Δ^, red solid bars, *n =* 4) mice. ^*^P < 0.05,^**^P < 0.01,^***^P < 0.001,^****^P < 0.0001.

Comparative expression analysis of KLF5 in multiple solid tumors and leukemia indicated that KLF5 expression was significantly decreased in leukemia when compared with other solid tumors, as analyzed in publicly available databases and summarized by the National Institutes of Health (http://cancergenome.nih.gov) (Supplementary Figure 1A). In addition, an analysis of a genome-scale shRNA screen of 501 cancer cell lines, revealed that five non-BCR-ABL B-ALL cell lines are not enriched for a dependency on KLF5, indicating that KLF5 does not score as an oncogenic- or tumor suppressor-dependency for non-BCR-ABL B-ALL (Supplementary Figure 1B) [20]. Interestingly, when grouped by mutation type, *KLF5* mRNA expression was significantly reduced in BCR-ABL1 B-ALL compared to all the other subtypes of pediatric ALL (Supplementary Figure 1C; *p* < 0.01). To validate these public expression datasets, we assessed the expression of *KLF5* in a set of human pro-B and pre-B ALL human cell lines harboring different mutations. We found *KLF5* mRNA expression decreased in BCR-ABL1 expressing cell lines compared with cell lines expressing other oncogene drivers that are known to transform in B-ALL, including those with *MLL* rearrangement or *TEL/AML1* translocations (Figure 1A–1B). The expression of KLF5 in CD34+/CD19+ cells from three specimens of normal and BCR/ABL1+ B-ALL adult BM was assessed by flow cytometry analysis. KLF5 expression in leukemic B-cell precursors was reduced by approximately 40% compared with normal B-cell precursors (Figure 1C and Supplementary Figure 1D).

### Forced expression of KLF5 results in increased apoptosis in imatinib-resistant Ph+ B-ALL

To determine whether loss of KLF5 was associated with important features of B-cell transformation, we tested the effects of forced overexpression of *KLF5* in BCR-ABL1 negative and positive B-ALL cell lines (Supplementary Figure 2A–2B). In BCR-ABL1+ cell lines BV-173, Z-119, NALM-1 and NALM-20 [21–24], KLF5 expression caused apoptosis (Figure 1D and Supplementary Figure 2C). However, in other non-BCR-ABL1 expressing cell lines, REH carrying the TEL-AML1 translocation, RS4;11 carrying the MLL-AF4 translocation [25, 26], or a BCR-ABL1+ cell line (SUP-B15) with pseudodiploidy and complex karyotype [27], forced expression of KLF5 did not induce apoptosis (Figure 1D). Z-119 and NALM-1 are cell lines that do not carry BCR-ABL1 mutations but are imatinib resistant [28, 29]. While imatinib, as expected, failed to induce apoptosis of these lines, the overexpression of KLF5 enhanced apoptosis 3-fold (Figure 1E). Expression of KLF5 in BV-173 cells, a cell line relatively sensitive to imatinib [30] did not enhance imatinib induced apoptosis (Figure 1E). Interestingly, forced expression of KLF5 did not alter B-ALL proliferation in any of the cell lines analyzed (Supplementary Figure 2D). These data, along with our published experience of detecting no effect of the gain-of-function of Klf5 on normal hematopoiesis, suggest that *KLF5* acts as a pro-apoptotic gene through a mechanism downstream of BCR-ABL1 signaling which may result in sensitization of imatinib resistant lymphoblastic cells.

### *Klf5* gene deletion enhances B-cell leukemogenesis *in vitro* and *in vivo*

We first tested whether KLF5 deficiency is associated with changes in B-cell differentiation. The pattern of differentiation of B-cell precursor/precursor subpopulations was assessed in the BM of *Vav1-Cre*; *Klf5*^*flox/flox*^ mice [31, 32] where the deficiency of Klf5 depends on the hematopoietic specific expression of Vav1 [33]. In these mutant mice, *Klf5* expression was found to be reduced by over 90% in B-cell precursors (Supplementary Figure 2E). *Klf5* is highly expressed in the B-cell precursor population compared with more primitive pre-proB cells and later precursor pre-B and surface IgM+ B cells of normal mouse BM (Supplementary Figure 2F). Loss of Klf5 in *Vav1-Cre*; *Klf5*^*flox/flox*^ BM did not impact normal B-cell precursor differentiation *in vivo*, as assessed by quantification of common lymphoid precursors (CLP), pre-pro B, pro-B, pre-B, and immature B, and mature B-cell populations in the BM of *Vav1-Cre*; *Klf5*^*flox/flox*^ mice (Supplementary Figure 2G–2L). Functional analysis of B-cell precursors in a colony-forming-cell assay was consistent with these findings (Supplementary Figure 2M). These data further support our previous report demonstrating the dispensable role of Klf5 in HSC and myeloid differentiation *in vivo* [31].

To determine whether KLF5 played a role in the pathogenesis of BCR-ABL1+ B-ALL, we tested the effect of *KLF5* deficiency in a retroviral transduction/transplantation murine model of p190-BCR-ABL B-ALL. Low-density bone marrow (BM) cells (LDBM) from *Vav1-Cre*; *Klf5*^*flox/flox*^ or WT mice were transduced with p190-BCR-ABL expressing bicistronic EGFP-expressing vectors. EGFP+ B-cell precursors (B220+dim/CD43+/IgM-) were sorted and cultured in the absence of stromal cell with rm-IL-7 and rm-SCF. At 8-days of culture, the *ex-vivo* expansion of p190-BCR-ABL+, *Klf5*^*∆/∆*^ B-cell precursors was 2.5- to 3.5-fold increased (Figure 1F–1G) with decreased apoptosis (Figure 1H).

In order to determine whether Klf5 deficiency impairs B-cell leukemogenesis once leukemia have been initiated, we generated *Mx1-Cre*;Klf5^flox/flox^ mice where the deficiency of Klf5 can be induced after transplantation [31] in recipient mice where leukemogenesis has already been initiated [34]. Transplantation of p190-BCR-ABL transduced *Mx1-Cre*;WT or *Mx1-Cre*;*Klf5*^flox/flox^ LDBM cells into lethally irradiated congenic CD45.1+ recipient mice resulted in the presence of over 90% engraftment as indicated by the expression of CD45.2+ hematopoietic cells and the similar levels of leukemic EGFP+ cells seen in peripheral blood by Day 18 post-transplantation in the recipient mice transplanted with *Mx1-Cre*;WT or *Mx1-Cre*;*Klf5*^flox/flox^ LDBM cells before administration of poly I:C (Supplementary Figure 3A). Poly I:C (5 mg/kg every other day) was administered intraperitoneally starting on Days 18 through 26 after transplantation, to induce interferon-inducible Mx1-Cre expression [31] resulting in the loss of *Klf5* expression in BM B-cell precursors from recipient mice of leukemic *Mx1-Cre*;*Klf5*^flox/flox^ BM cells (Figure 1I), and the median survival of mice transplanted with *Mx1-Cre*;*Klf5*^flox/flox^ BM post-poly I:C administration was reduced by ∼50% (from 48 to 24 days; Figure 1J). The leukemic B-cell precursors from leukemic *Mx1-Cre*; *Klf5*^flox/flox^ BM cells also showed decreasing apoptosis (Figure 1K and Supplementary Figure 3B). Levels of circulating leukemic cells (Supplementary Figure 3C), BrdU incorporation in B-cells (Supplementary Figure 3D) and B-cell differentiation *in vivo* (Supplementary Figure 3E) were not affected, indicating that Klf5 deficiency predominantly affects the survival of B-cell precursors. Altogether, these data indicate that Klf5 is a tumor suppressor through the promotion of apoptosis of p190-BCR-ABL B-ALL.

### Klf5 rescues primary CFU formation and survival *in vitro* and *in vivo* in Klf5 deficient leukemic precursors

**Figure 2:**
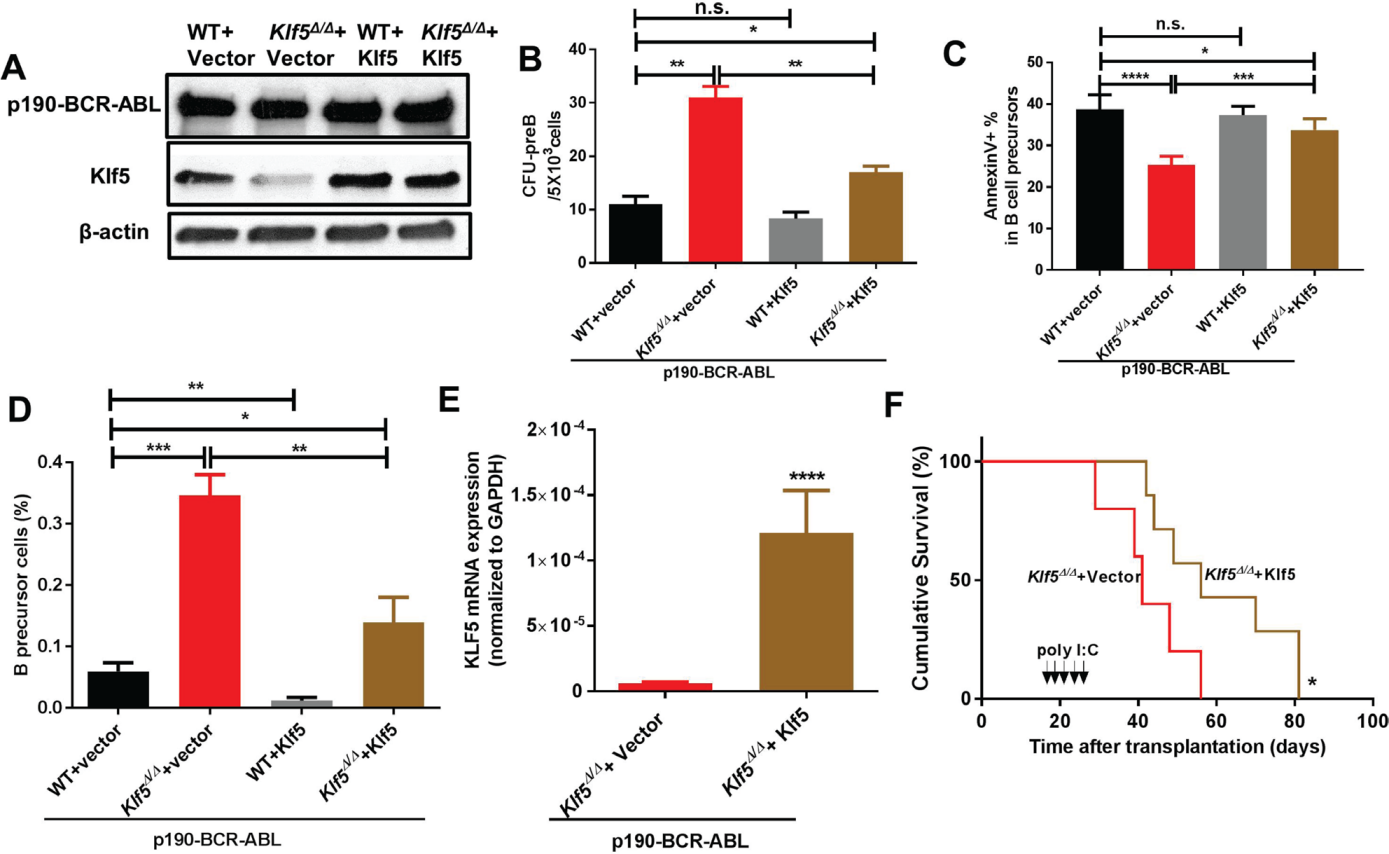
Klf5 rescues Klf5-deficient, p190-BCR-ABL leukemogenesis and leukemic precursor survival (**A**) Representative example of immunoblot showing p190-BCR-ABL and Klf5 expression in B-cell precursors generated from *Vav1-Cre*; WT (WT) or *Vav1-Cre*; *Klf5*^*flox/flox*^ (*Klf5*^Δ/Δ^) BM co-transduced with p190-BCR-ABL and either mKlf5 (WT + Klf5 or *Klf5*^Δ/Δ^ + Klf5) or empty vectors (WT + vector or *Klf5*^Δ/Δ^ + vector). β-actin expression analysis from total lysate was used as a loading control. (**B**) CFU-preB content of sorted B-cell precursors from *Vav1-Cre*; WT (WT) or *Vav1-Cre*; *Klf5*^*flox/flox*^ *(Klf5*^Δ/Δ^*)* BM co-transduced with p190-BCR-ABL and either mKlf5 (WT + Klf5 or *Klf5*^Δ/Δ^ + Klf5) or empty vectors (WT + vector or *Klf5*^Δ/Δ^ + vector). Data derived from two independent experiments. Each experiment was performed in triplicate and data are given as mean ± SEM. (**C**) Apoptosis assessed as fold-increase of annexin V+ cell percentage over control (WT transduced with a mock vector) of B-cell precursors generated from *Vav1-Cre*; WT (WT) or *Vav1-Cre*; *Klf5*^*flox/flox*^ (*Klf5*^Δ/Δ^) BM co-transduced with p190-BCR-ABL and either mKlf5 (WT + Klf5 or *Klf5*^Δ/Δ^ + Klf5) or empty vectors (WT + vector or *Klf5*^Δ/Δ^ + vector). Data was derived from two independent experiments. Each experiment was performed in triplicate and data are given as mean ± SEM. (**D**) Frequency of *ex-vivo* generated B-cell precursors generated from *Vav1-Cre*; WT (WT) or *Vav1-Cre*; *Klf5*^*flox/flox*^ (*Klf5*^Δ/Δ^) BM LSK cells co-transduced with p190-BCR-ABL and either mKlf5 (WT + Klf5 or *Klf5*^Δ/Δ^ + Klf5) or empty vectors (WT + vector or *Klf5*^Δ/Δ^ + vector). Data derived from two independent experiments. Each experiment was performed in triplicate and data are given as mean ± SEM. (**E**) *Klf5* mRNA expression in p190-BCR-ABL+ B-cell precursors from *Klf5*^Δ/Δ^ mice expressed with either empty vector (*n =* 5) or Klf5 (*n =* 6). Values are given as mean ± SD. (**F**) Survival of mice transplanted with 1 × 10^6^ *Mx1-Cre*; *Klf5*^*flox/flox*^ BM cells co-transduced with p190-BCR-ABL and either mKlf5 (*Klf5*^Δ/Δ^ + Klf5; brown line, *n =* 5) or empty(*Klf5*^Δ/Δ^ + vector; red line, *n =* 7). ^*^P < 0.05 (Log-rank test). ^*^P < 0.05, ^**^P < 0.01,^***^P < 0.001,^****^P < 0.0001.

Lentiviral expression of Klf5 (Figure 2A) rescued the CFU-pre B colony formation and apoptosis of *Klf5*^*∆/∆*^ leukemic B-cell precursors (Figure 2B–2C and Supplementary Figure 3F–3G), as well as the overall number of leukemic B-cell precursors (Figure 2D). This suggests that Klf5 suppressor activity is specifically affecting leukemic B-cell progenitors/precursors. *Mx1-Cre*; *Klf5*^*flox/flox*^ LDBM cells expressing p190-BCR-ABL were transduced with an empty vector or mKlf5 followed by transplantation into lethally irradiated recipient mice. After *Klf5* gene deletion induction (Figure 2F, arrows), we found that Klf5 expression (Figure 2E) extended the survival of *Klf5*^*∆/∆*^ leukemic mice (Figure 2F), which supports the role of Klf5 as a tumor suppressor in p190 BCR-ABL transformed B-ALL.

### Klf5 regulates glutathione-S-transferase transcription influencing oxidative metabolism

**Figure 3:**
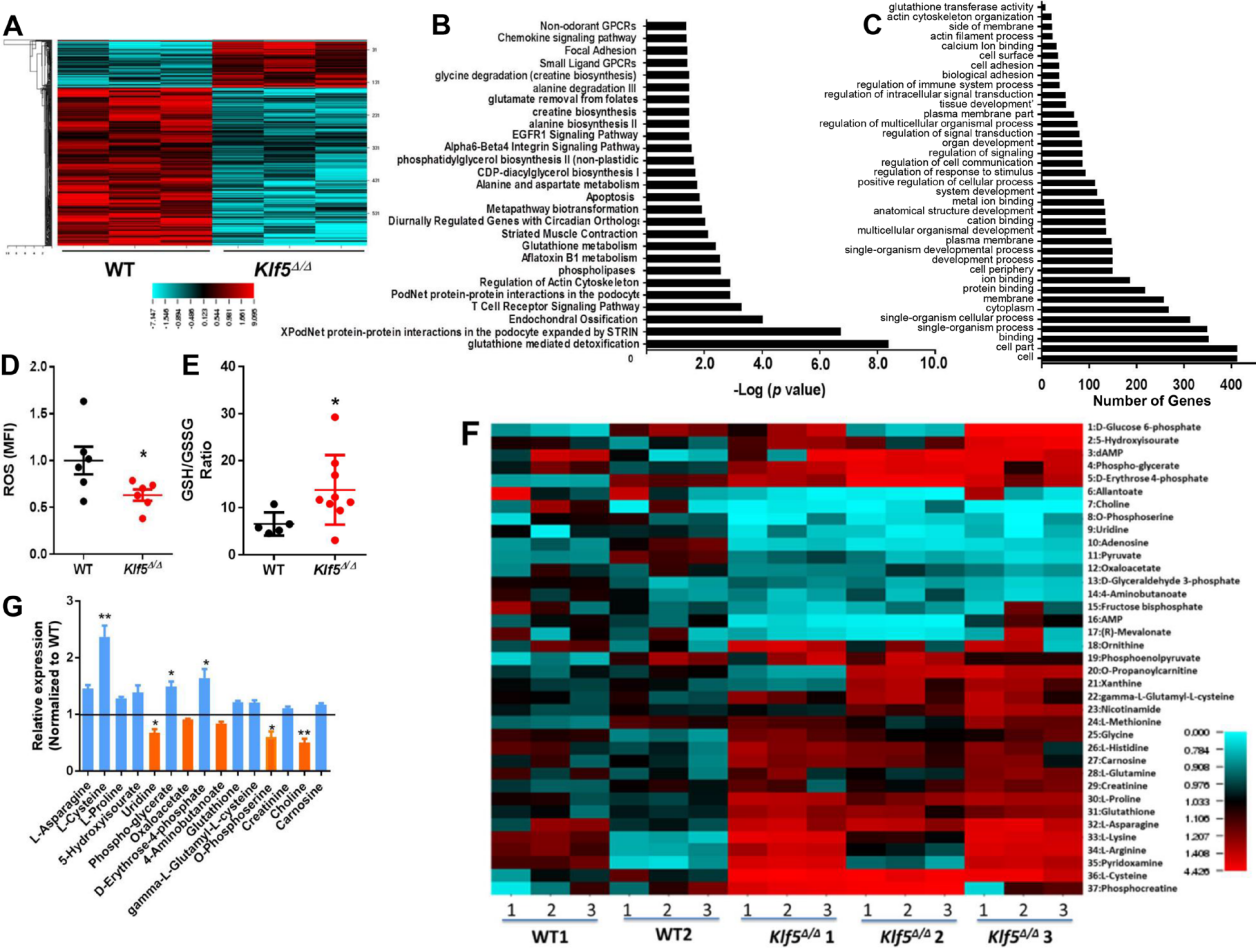
Decreased GST expression, reduced ROS, increased GSH/GSSG ratio and dysregulated metabolome in *Klf5*^Δ/Δ^ leukemic B-cell precursors (**A**) Heat map of normalized RNA-seq data showed 140 upregulated genes and 489 downregulated genes in *Klf5*^Δ/Δ^ (*n =* 3) compared with WT (*n =* 3) leukemic B-cell precursors (cut-off > 2.0 fold changes, *P* < 0.05). (**B**) Pathway analysis of the comparative transcriptome of leukemic WT and *Klf5*^Δ/Δ^ B-cell precursors. Metabolic pathways are highlighted in bold. *n =* 3 replicates, cut off > 2.0 fold changes, *P* < 0.05. (**C**) Gene ontology (GO) analysis of the comparative transcriptome of leukemic WT and *Klf5*^Δ/Δ^ B-cell precursors. The glutathione transferase pathway is highlighted in bold (6 genes, validated in Figure 4A). *n =* 3 replicates, cut off > 2.0 fold changes, *P* < 0.05. (**D**) Reactive oxygen species levels in leukemic B-cell precursors from Mx1-Cre; WT (WT) and Mx1-Cre; *Klf5*^*flox/flox*^ (*Klf5*^Δ/Δ^) BM. Each sample was measured in triplicate. (**E**) GSH/GSSG ratios of leukemic B-cell precursors from Mx1-Cre; WT (WT) and Mx1-Cre; *Klf5*^*flox/flox*^ (*Klf5*^Δ/Δ^) BM. Each sample was measured in triplicate. (**F**) Heat map of comparative metabolome of B-cell precursors with significant differences between p190-BCR-ABL+ leukemic Mx1-Cre; WT (WT, *n =* 2) and Mx1-Cre; *Klf5*^*flox/flox*^ (*Klf5*^Δ/Δ^, *n =* 3) B-cell precursors. Each sample was measured in triplicate for each mouse derived specimen. (**G**) Fold change in metabolite concentrations of *Klf5*^Δ/Δ^ B-cell precursors with significant differences (*p* < 0.05) compared to WT B-cell precursors. Blue bars denote increased levels and orange bars denote decreased levels in *Klf5*^Δ/Δ^ B-cell precursors. ^*^P < 0.05.

KLF4, a related member of the KLF family of transcription factors, has been previously identified as a tumor suppressor in B-cell lymphoproliferative disorders [35]. KLF4 is known to block transformation by BCR-ABL1, to induce arrest and apoptosis in the G1 phase of leukemic B-lineage cells, and to deplete leukemic pre-B cells *in vivo* through an unclear molecular mechanism [35]. To identify whether KLF5 acts similarly to KLF4, we first compared the transcriptome of BM leukemic WT and *Klf5*^*∆/∆*^ B-cell precursors (Gene Expression Omnibus (GEO) accession number GSE 115919). Using an unsupervised approach, we found that the expression of 140 genes was increased, and was repressed in 489 genes in leukemic *Klf5*^Δ/Δ^ B-cell precursors (Figure 3A; Supplementary Data). Deletion of *Klf5* influenced the expression of B-lineage survival genes including survivin (*Birc5*) [36], *Fas* [37, 38], *Tnfsf10* [39], *Irf7* [40] and *Casp4* [41] (Supplementary Figure 4A–4E). The *Ikzf1, Cbx5, Hmgb3, Ebf1* and *Pax5* genes, all of which have been implicated in B-cell differentiation and associated with the progression of BCR-ABL1 B-ALL, were not affected by the loss of Klf5 in leukemic B-cell precursors (Supplementary Figure 4F–4J). The expression levels of other members of the Klf family of transcriptional factors did not change significantly (Supplementary Figure 4K). Nor did the expression of self-renewal transcription factors *Klf4* and *Tal1* (Supplementary Figure 4L–4M) and cell cycle related genes, such as *Ccnd1, Cdkn1a, Cdkn1b, Cdkn1c, Cdkn2a*, and *Cdkn2b* (Supplementary Figure 4N–4S), supporting the concept that Klf5 effect on the survival of precursors is not mediated by compensatory regulation of other transcriptional networks. Genes implicated in apoptosis, glutathione-mediated detoxification, lipid related metabolism and amino acid metabolism pathways, and transformation-associated cytoskeletal regulation, were found to be significantly altered in Klf5-deficient B-cell precursors (Figure 3B–3C). Because these pathways play an important role in modulating the biological effects of carcinogens and in metabolizing a broad range of ROS and xenobiotics, we measured ROS and the ratio of the reduced and oxidized forms of glutathione dioxide (GSH/GSSG) as indicators of the cellular oxidative stress in WT and *Klf5*^Δ/Δ^ leukemic B-cell precursors. ROS levels were decreased in Klf5-deficient leukemic precursors and the GSH/GSSG ratio was increased (Figure 3D–3E) indicating that Klf5 functions as a pro-oxidant in leukemic B-cell precursors. The metabolic profile of B-cell precursors from the WT and *Klf5*^Δ/Δ^ groups identified 37 metabolites whose levels were different (Figure 3F–3G). These metabolites participate in glycolysis, the pentose phosphate pathway, glycerophospholipid biosynthesis, serine biosynthesis and one-carbon metabolism, the tricarboxylic acid and urea cycles, the GABA shunt, carnitine and fatty acid metabolism, GSH homeostasis, redox, and the arginine and proline metabolism pathways. Among them, the anti-oxidants and anti-oxidant precursors L-asparagine, L-cysteine, L-proline, 5-hydroxyisourate, phospho-glycerate, D-erythrose-4-phosphate, glutathione, gamma-L-glutamyl-L-cysteine, creatinine, and carnosine were increased, while the pro-oxidants or pro-oxidant precursors uridine, oxaloacetate, 4-aminobutanoate, O-phosphoserine, and choline were significantly decreased in Klf5 deficient leukemic B-cell precursors. Altogether, these data indicate that *Klf5*^Δ/Δ^ leukemic B-cell precursors acquire an antioxidant state associated with increased survival.

### *Gstm1* is a direct target of Klf5 and is rescued by forced expression of Klf5 or Gstm1

**Figure 4:**
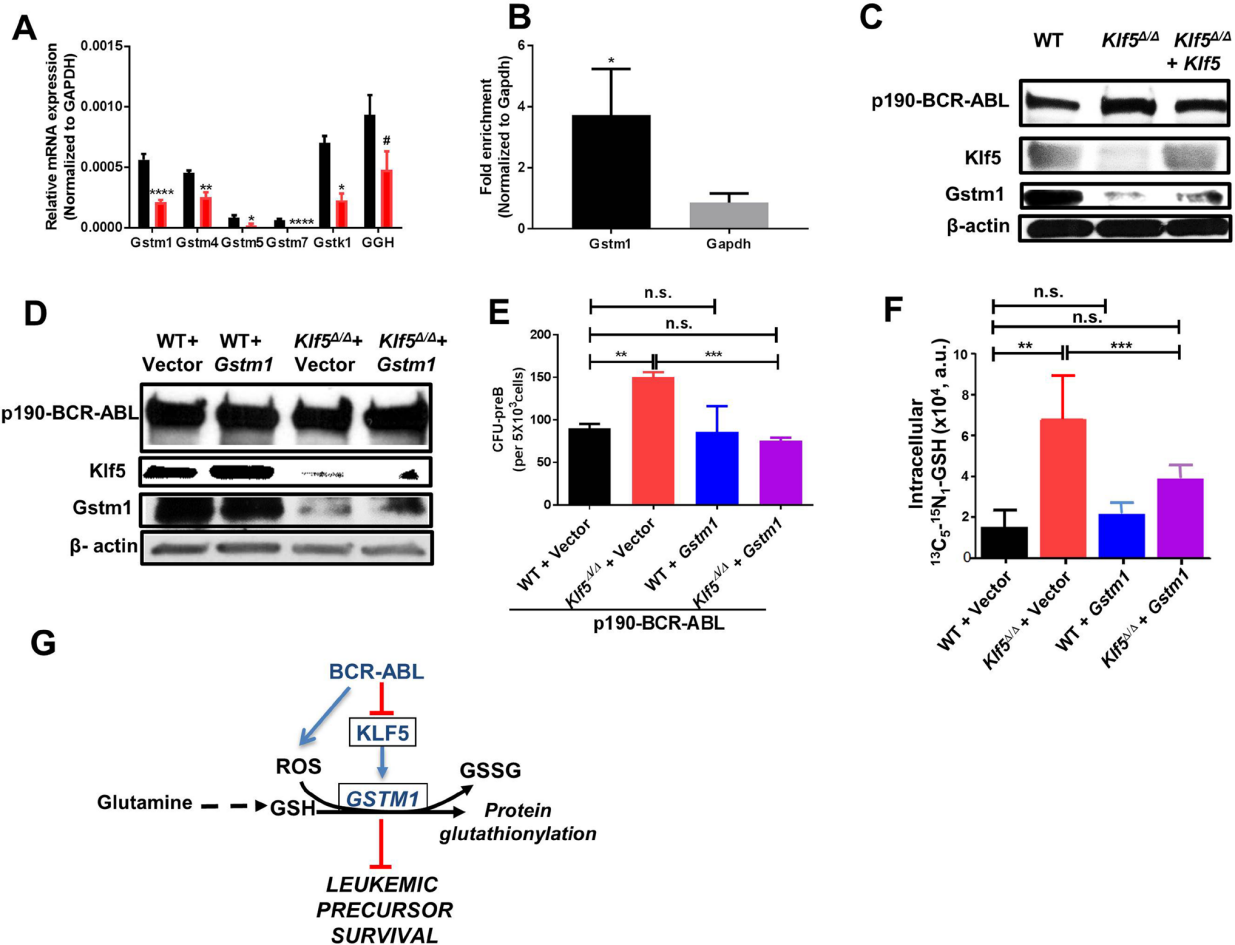
Glutathione-S-transferase activity restores tumor promotion activity of Klf5-deficient B-cell precursors (**A**) Q-PCR validation of GST genes (from Figure 3C) (black bar: WT; red bar: *Klf5*^Δ/Δ^*)*. Each experiment was performed in triplicate and data are given as mean ± SEM. (**B**) ChIP-qPCR showed fold enrichment of Gstm1 normalized to negative gene control glyceraldehyde 3-phosphate dehydrogenase (GAPDH) (both normalized to related IgG previously). Data are presented as mean ± SD of three independent replicates. (**C**) Representative example of immunoblot for p190-BCR-ABL, Klf5 and Gstm1 expression in leukemic BM B-cell precursors from chimeric mice transplanted with *Mx1-Cre*; WT transduced with a mock vector (WT), *Mx1-Cre*; *Klf5*^*flox/flox*^ transduced with a mock-vector (*Klf5*^Δ/Δ^*)* or *Mx1-Cre*; *Klf5*^*flox/flox*^ transduced with vector expressing mKLF5 (*Klf5*^Δ/Δ^ *+ Klf5)*. β-actin expression analysis from total lysate was used as a loading control. Two independent experiments were performed. (**D**) Representative example of immunoblot for p190-BCR-ABL, Klf5 and Gstm1 expression in leukemic B-cell precursors from *in vitro* cultured *Vav1-Cre*; WT (WT) or *Vav1-Cre*; *Klf5*^*flox/flox*^ (*Klf5*^Δ/Δ^) BM co-transduced with p190-BCR-ABL and either mGstm1 (WT + Gstm1 or *Klf5*^Δ/Δ^ + Gstm1) or empty vectors (WT + vector or *Klf5*^Δ/Δ^ + vector). β-actin expression analysis from total lysate was used as a loading control. Two independent experiments were performed. (**E**) Frequency of CFU-preB from B-cell precursors from *in vitro* cultured *Vav1-Cre*; WT (WT) or *Vav1-Cre*; *Klf5*^*flox/flox*^ (*Klf5*^Δ/Δ^) BM co-transduced with p190-BCR-ABL and either mGstm1 (WT + Gstm1 or *Klf5*^Δ/Δ^ + Gstm1) or empty vectors (WT + vector or *Klf5*^Δ/Δ^ + vector). Each experiment was performed in triplicate and data are given as mean ± SEM. (**F**) Metabolite tracking of the change in levels of ^13^C_5_^15^N_1_-glutathione in Mx*1-Cre*; WT (WT) and *Mx1-Cre*; *Klf5*^*flox/flox*^ (*Klf5*^Δ/Δ^*)* leukemic B-cell precursors after transduction with Gstm1 (WT + *Gstm1* or *Klf5*^Δ/Δ^ *+ Gstm1)* or empty vectors (WT + Vector or *Klf5*^Δ/Δ^ *+* Vector) and cultured for 24 hours. Each experiment was performed in triplicate and data are given as mean ± SD. (**G**) Schema to illustrate the putative mechanism regulated by Klf5 and Gstm1. ^#^*P* = 0.06; ^*^P < 0.05, ^**^P < 0.01, ^****^P < 0.0001.

The gene ontology (GO) and pathway analysis of the RNA-seq data both pointed to dysregulation of glutathione–mediated detoxification and glutathione metabolism, which was greatest in the differential transcriptional regulation of the gene *Gstm1*. We used quantitative PCR (Q-PCR) to validate the expression of the genes seen in the RNA-Seq data (Figure 4A). The *Gstm1* promoter region contains two consensus motifs which predict binding by KLF family members. Chromatin immunoprecipitation (ChIP) followed by Q-PCR demonstrated the binding of Klf5 to the *Gstm1* promoter (Figure 4B) in leukemic B-cell precursors. The expression of Klf5 in *Klf5*^Δ/Δ^ leukemic B-cell precursors increased the expression of *Gstm1* (Figure 4C). Remarkably, expression of Gstm1 (Figure 4D) restored the CFU-preB colony formation of Klf5-deficient B-cell precursors to WT levels (Figure 4E), indicating that Klf5 and its downstream target Gstm1 reduce the leukemogenic potential of B-cell precursors.

### Glutamine dependent glutathione utilization in leukemic B-cell precursors depends on the axis Klf5/Gstm1

Because the ROS levels, GSH/GSSG ratio and metabolomics data indicated that Klf5 deficient leukemic B-cell precursors acquired an antioxidant state with increased GSH, we hypothesized that Klf5 dependent Gstm1 expression may control glutathione-mediated detoxification. Since glutaminolysis is a major path associated with glutathione biosynthesis, we performed a metabolic tracing by adding ^13^C_5_^15^N_2_ glutamine into the cell culture media and assaying the steady-state levels of the heavy glutathione isotopologue M+5+1 (as synthesized from incorporation of ^13^C^15^N-glutamine-derived glutamate into the GSH tripeptide at 24 h from incubation with the stable isotope tracer; Figure 4F). This data indicates that the expression of Gstm1 restores the increased glutaminolysis flux to glutathione to normal levels in *Klf5*^*∆/∆*^ B-cell precursors (Figure 4G). Together with the functional restoration of *Klf5*^*∆/∆*^ B-cell precursor apoptosis, this data indicates that Gstm1 is a functionally relevant target of Klf5 in the maintenance of GSH levels and protein glutathionylation in leukemic B-cell precursors. Altogether, these results indicate that the Klf5 axis acts as a tumor suppressor by promoting GSH depletion and glutathione-S-transferase mediated glutathionylation (Figure 4G).

## DISCUSSION

The KLF transcription factor family has both essential and redundant functions. Klf5, similar to the other 16 members of the KLF family, contains three zinc-finger domains that function in DNA binding. In hematopoiesis, KLF proteins have been shown to play redundant roles. For instance, Klf1- and Klf2-dependent globin expression have been shown to compensate for each other in primitive erythropoiesis, but Klf1 has a distinct role in the expression of β-globin in definitive erythropoiesis [42]. KLF5, one of the members of the KLF family, is highly expressed in hematopoietic stem cells (HSC) and in differentiated myeloid cells [43]. Klf5 expression, controlled by Klf4, appears to control the granulocyte-macrophage differentiation of committed myeloid progenitors/precursors in response to granulocyte colony-stimulating factor-signaling [44, 45] and to regulate monocyte/macrophage differentiation and activation [46, 47]. We previously reported that Klf5 controls BM adhesion, retention, homing, endosteal lodging and engraftment of HSC and progenitors, but is not essential for HSC self-renewal [31]. The mechanism of this activity is its transcriptional activation of Rab GTPases, which in turn controls the endosome recycling of β_1_/β_2_ integrins that is required for adhesion and homing of HSC.

Both Klf5 and Klf4 are highly expressed in different lymphoid compartments and may act as lymphoproliferative suppressors [35]. While similar in structure, KLF5 and KLF4 are distinctly developmentally and phylogenetically regulated [48], suggesting the existence of non-conserved functions. The role of Klf4 in the processes of leukemogenesis in B-ALL and T-cell ALL has been carefully studied [35, 49]. Klf4 was proposed to repress B-lymphoid leukemogenesis through arrest in the G1 phase of the cell cycle and apoptosis. Transformed pre-proB cells expressing Klf4 display increased expression of p21CIP and decreased expression of c-Myc and cyclin D2. Klf4-mediated death, but not cell-cycle arrest, can be rescued by Bcl-X_L_ overexpression. In addition, Klf4 can repress T-cell leukemogenesis through aberrant activation of the mitogen-activated protein kinase kinase, MAP2K7, and the downstream c-Jun NH_2_-terminal kinase (JNK) pathway. KLF5 involvement in lymphoid leukemogenesis is however unclear. Zhu *et al.* proposed that KLF5 acts as a lymphoblastic leukemia oncogene by interacting with p53 to regulate survivin expression [18]. Herein, we report that BCR-ABL1+ B-ALL has a strikingly reduced expression of KLF5, which is supported by the reported hypermethylation of the *KLF5* promoter in patient-derived BCR-ABL1+ leukemic cells [19], suggesting that KLF5 may play preferentially a role as tumor suppressor in BCR-ABL1+ B-ALL.

Our data indicate that KLF5-expression increased apoptosis of leukemic B-cell precursors, particularly in imatinib-resistant Ph+ B-ALL cells without affecting the differentiation of either leukemic or normal B-cell lymphopoiesis, suggesting that KLF5 may be a possible target in overcoming the resistance to ABL TKI seen in a fraction of Ph+ B-ALL patients. We found that exogenous expression of Klf5 resulted in reduced B-cell precursor colony formation and reduced cell survival of *Klf5*^*∆/∆*^ leukemic precursor cells *in vitro* and *in vivo*. Klf5 deficiency acted as an antioxidant, inhibited apoptotic transcriptional programs, including pro-apoptosis gene *Fas, Tnfsf10, Irf7* and *Casp4,* and induced the anti-apoptosis gene *Birc5*, a finding opposite that to reported for Klf5 in other types of B-ALL [18].

In our data set, we found that GSTM1, a critical enzyme involved in the detoxification process of oxidative species and associated with poor-prognosis pediatric B-ALL [50–54], is a direct transcriptional target of KLF5 and is decreased in *Klf5*^*∆/∆*^ B-cell precursors. We hypothesized that in BCR-ABL1+ B-ALL, KLF5 may function as a negative regulator of oxidative stress through direct regulation of GSH metabolism. We found that forced Gstm1 expression repressed and restored B-cell precursor colony formation; Klf5 deficiency increased glutathione biosynthesis, which was restored to normal levels by forced expression of Gstm1; and finally, the expression of Klf5 or Gstm1 downregulated glutathione biosynthesis to the basal levels seen BCR-ABL expressing leukemic precursors.

Oxidative stress results from an imbalance between oxidant and anti-oxidant levels within the cell. BCR-ABL is a major inducer of oxidative stress through multiple pathways, which ultimate result in increased genomic instability, especially in imatinib-resistant leukemia [55–57]. When oxidative stress increases beyond a level manageable for the cell, oxidative damage is sustained, which leads to cellular death [58]. This effect can be exploited therapeutically, and some pro-oxidants are effective in eliminating imatinib-resistant leukemic residual cells [59, 60]. GSH is the most abundant non-enzymatic antioxidant molecule in the cell and is essential for cell survival and redox homeostasis [61]. Modifications in GSH metabolism have been described in many tumor types and are associated with oncogenesis (reviewed in [62]) as they prevent spontaneous cancer cell apoptosis or that triggered by chemotherapy [63–65]. Moreover, high GSH levels induce resistance to TKI through a diversity of postulated mechanisms [54, 66–70].

Glutamine is the most abundant amino acid in plasma and in intracellular pools, being consumed at significantly higher rates than other amino acids by tumor cells, and it is essential for GSH biosynthesis [71]. Glutamate and cysteine are combined into gamma-glutamyl-cysteine which is then combined with glycine by GSH synthetase to generate GSH. GSH levels depend on the activity of the enzymes regulating GSH utilization in protein glutathionylation, a basic mechanism controlling protein degradation and endoplasmic stress response [72]. Decreased GST may reduce the consumption of GSH and lead to higher levels of GSH. Protein glutathionylation depends largely on GST activity. Interestingly, knocking-out Klf5 inhibited the expression of genes involved in glutathione-mediated detoxification, specifically those ones involved in GST activity. The loss of Klf5 decreased oxidative stress, increased regeneration of GSH and inhibited apoptosis.

In conclusion, Klf5 is efficiently downregulated in imatinib-resistant p190-BCR-ABL B-ALL leukemic cells and it acts as a negative regulator of the anti-oxidant glutathione. Klf5 is a negative regulator of leukemic B-cell precursor survival. Expression of Klf5 reduces leukemic B-cell precursor survival, and its role in controlling anti-oxidant glutathione levels depends on its transcriptional activating role on the expression of glutathione transferase genes. The leukemic suppressor effect of Klf5 on precursor survival can be restored by downstream overexpression of Gstm1.

## MATERIALS AND METHODS

### Animals

Our group has reported the generation and validation of Mx1-Cre and Vav1-Cre transgenic mice driving the deletion of Klf5 [31, 32]. C57Bl/6J mice (Jackson Laboratories) were used as recipients.

### Patient samples and cell lines

Normal BM CD34+ cells, and B-ALL mononuclear cells (BCR-ABL1+) specimens were obtained under Institutional Review Board-approved protocols and donor informed consent from Cincinnati Children’s Hospital Medical Center. B-ALL cell lines REH, RS4;11, SEM, KOPN8, RCH-ACV, NALM-1, SD-1, BV-173, SUP-B15, and NALM-20 were purchased from American Tissue Culture Collection (ATCC, Manassas, VA) or the Deutsche Sammlung von Mikroorganismen und Zellkulturen (DSMZ, Braunschweig, Germany). Z-181 and Z-119 were kindly provided by Dr. Zeev Estrov (MD Anderson, Houston, TX). REH, KOPN8, RCH-ACV, SD-1, Z-181, BV-173, Z-119, NALM-1, and NALM-20 were cultured in RPMI-1640 medium (Thermo Fisher Scientific, Waltham, MA) containing 20% fetal bovine serum (FBS) (Atlanta Biologicals, Flowery Branch, GA), 2 mM L-glutamine (Thermo Fisher Scientific), 100 U/ml penicillin and 100 μg/mL streptomycin (Thermo Fisher Scientific). RS4;11 was cultured in alpha-MEM (Minimum Essential Medium Eagle - alpha modification; StemCell Technologies, Vancouver, Canada) containing 20% FBS, 2 mM L-glutamine, 100 U/mL penicillin and 100 μg/mL streptomycin. SEM was cultured in Iscove’s Modified Dulbecco’s Medium (IMDM, Thermo Fisher Scientific) containing 20% FBS, 2 mM L-glutamine, 100 U/mL penicillin and 100 μg/mL streptomycin. SUP-B15 was cultured in McCoy’s 5A medium (Thermo Fisher Scientific) containing 20% FBS, 2 mM L-glutamine, 100 U/mL penicillin and 100 μg/mL streptomycin. In some experiments, imatinib mesylate (LC laboratories, Woburn, MA) was added to the culture medium.

### Viral transduction

Retroviral and lentiviral transduction have been described previously [73]. In brief, mouse low-density (LD) bone marrow (BM) (LDBM) cells or Lineage-/c-kit+/Sca1+ (LSK) cells were transduced with bicistronic retroviral vectors (MIEG3) encoding the BCR-ABL1 isoform p190 in the presence of 20 ng/mL of recombinant mouse IL-7 (PeproTech, Rocky Hill, NJ), 10 ng/mL of recombinant mouse SCF (PeproTech), and the recombinant fragment of fibronectin, CH296 (RetroNectin, Takara Bio Inc.) for 16 hours at 37° C. For the Klf5 or Gstm1 expression, lentiviral particles encoding either full-length mKlf5 cloned in a pCDH1-MCS1-EF1-copGFP vector (System Biosciences, Palo Alto, CA), mGstm1 (GeneCopoeia, Catalog #: EX-Mm02884-Lv166) or related empty vectors, were used for stable expression in p190-BCR-ABL+ B-cell lymphoid precursors. B-ALL cell lines were transduced with lentiviral particles expressing either full-length hKLF5 cDNA (GeneCopoeia, Catalog #: EX-T1795-Lv166) or the control bicistronic mCherry-expressing empty vector driven by the promoter EF1 at a multiplicity of infection of 10 for 6 hours at 37° C.

### Transplantation of transduced leukemic cells

We have previously reported our protocol of transduction/transplantation of leukemic BM cells [73]. In brief, LDBM cells from Mx1-Cre; WT or Mx1-Cre; Klf5^flox/flox^ littermates were isolated and transduced with a retroviral bicistronic vector encoding p190-BCR-ABL with enhanced green fluorescent protein (EGFP) as a reporter. For the Klf5 *in vivo* rescue experiments, lentiviral particles encoding either full-length mKlf5 cDNA cloned in a pCDH-EF1-MCS-IRES-RFP vector (System Biosciences), or the empty vector, were used for stable expression in p190-BCR-ABL+ B-cell lymphoid precursors. Transduced LDBM cells (1 × 10^6^) were intravenously transplanted into lethally (7 + 4.75 Gy) irradiated congenic CD45.1+ mice. The multiplicity of infection of p190-BCR-ABL was kept low to allow only one copy of BCR-ABL per transduced cell, and the multiplicity of infection of lentiviral mKlf5 was kept as 10. Peripheral blood was collected through retro-orbital puncture on Day 18 to test the engraftment of donor cells. Mice were then administered poly I:C intraperitoneally (5 mg/kg) every other day for a total of five times to delete the Klf5 gene. Mice were sacrificed when they showed signs of extreme hunched posture with labored breathing and/or symptoms of moribund or rear leg paralysis were noted. BM and spleens were dissociated and processed for flow cytometric analysis.

### Flow cytometry analysis and cell sorting

LDBM cells were obtained after centrifugation in Histopaque-1083 (Sigma, Saint Louis, MO). Transduced LDBM cells or harvested leukemic BM cells from B-ALL mice were purified for early B-cell precursors for further *in vitro* analysis. Early B-cell precursors were defined as EGFP+/B220+dim/CD19+/CD43+/membrane IgM-, and isolated by FACS (FACS Aria II, Becton Dickinson (BD), San Jose, CA) under sterile conditions. LDBM cells were stained with biotinylated lineage markers (CD45R (B220; clone RA3-6B2), Gr-1 (Ly-6G and Ly-6C; clone RB6-8C5), CD4 (L3T4; clone RM4-5), CD8a (Ly-2; clone 53-6.7), CD3e (clone 145-2C11), CD11b (clone M1/70), and Ter119 (clone Ly-76), streptavidin APC-Cy™7, allophycocyanin (APC)-conjugated c-kit (clone 2B8) and phycoerythrin (PE)-Cy7-conjugated Sca-1 (clone D7) antibodies for LSK cell population, and sorted (FACS Aria II). Commercially available fluorochrome-conjugated anti-mouse or anti-human antibodies were used (all from BD Pharmingen, San Jose, CA), including B220 (CD45R/B220, clone RA3-6B2), CD11b (clone M1/70), CD3e (clone 145-2C11), CD43 (clone S7), CD93 (clone AA4.1), CD19 (Clone 1D3), IgM (clone R6-60.2), mouse anti-human CD34 (clone 581), and mouse anti-human CD19 (clone SJ25C1). Analysis was performed using a flow cytometer (FACS Canto, BD). KLF5 expression of CD34+CD19+ cells from normal donor and BCR-ABL1+ patients was carried out by flow cytometry analysis. Samples were prepared according to manufacturer’s protocol using BD Pharmingen Transcription Factor Buffer Set. Anti-KLF5 antibody (1:2000) (Catalog # 61099, Active Motif, Carlsbad, CA) and FITC Goat Anti-Rabbit IgG (BD Pharmingen, Catalog # 554020) were used.

### B-cell lineage colony-forming unit (CFU-preB) assay

B-cell lineage colony-forming units (CFU-preB) were quantified after 9 days of culture of whole BM cells or after 7 days culture of sorted p190 BCR-ABL expressing B-cell precursors in M3134 methylcellulose (StemCell Technologies) supplemented with 30% FBS (for mouse B lymphoid colony forming cells; StemCell Technologies), 2 mM L-glutamine, 100 U/ml penicillin and 100 μg/mL streptomycin, 100 μM β-mercaptoethanol (Fisher-Scientific), 1% BSA (Roche Diagnotics), 20 ng/mL of recombinant mouse IL-7 (PeproTech), and 100 ng/mL of recombinant mouse SCF (PeproTech) [73].

### Cell cycle and apoptosis assays

Cell cycle was analyzed by 5-bromo-2’-deoxyuridine (BrdU) incorporation on gated red fluorescent (mCherry +) ALL cell line cells or on gated EGFP+ B-cell precursors following the manufacturer’s protocol (BrdU Flow Kit, BD Pharmingen). Apoptosis was evaluated by flow cytometric determination of annexin V (BD Pharmingen) binding on gated mCherry+ B-ALL cell lines or on gated EGFP+ primary or *ex-vivo* generated B-cell precursors. For live cell gating analysis, we used 7-aminoactinomycin D (Invitrogen, ThermoFisher Scientific).

### Quantitative RT-PCR analysis

Total mRNA was isolated from ALL cell line cells or p190-BCR-ABL expressing mouse B-cell precursors according to the manufacturer’s protocol (RNeasy Micro Kit, Qiagen Sciences Inc., Germantown, MD). mRNA was reverse-transcribed using TaqMan^®^ Reverse Transcription Reagents (Applied Biosystems, Thermo Fisher Scientific), followed by amplification of cDNA (Taqman Universal PCR master mix, Applied Biosystems). Cycle threshold (Ct) values of individual genes were normalized to glyceraldehyde 3-phosphate dehydrogenase (GAPDH) and values calculated using the 2^−ΔCt^ method.

### Western blot assays

FACS sorted purified B-cell precursors or ALL cell line cells were lysed in radioimmunoprecipitation assay (RIPA) buffer and extracts were electrophoresed on SDS-PAGE. For immunoblotting, separated proteins were transferred to a PVDF membrane. The membrane was then blocked with 5% non-fat milk in tris-buffered saline (TBS) for one hour at room temperature (RT). A guinea pig anti-Klf5 antibody [31] (1:4000) was used for mouse samples, anti-KLF5 antibody (Abcam, Cambridge, MA, Catalog # ab24331,1:1000) for human ALL cell line samples, c-Abl antibody (Cell Signaling Technology, Catalog # 2862, 1:1000), Gstm1 antibody (Santa Cruz Biotechnology, Catalog # sc-133641, 1:500) was added separately and incubated overnight at 4°C. Mouse anti-β-actin antibody (Sigma) was added as a loading control. The filters were washed, incubated with a secondary anti-mouse, rabbit or guinea pig HRP-conjugated antibody for one hour at room temperature and the bands were visualized using enhanced chemiluminescence (Amersham ECL, GE Healthcare).

### Metabolomics studies

Targeted metabolomics analysis was performed as previously reported [74]. Cultured p190-BCR-ABL+ leukemic WT + vector, WT + Gstm1, *Klf5*^Δ/Δ^ + vector and *Klf5*^Δ/Δ^ + Gstm1 groups were labeled with 4 mM L-glutamine (^13^C_5_, 99%; ^15^N_2_, 99%, Cambridge Isotopes). At time points 0 and 24 hours, the cells were collected and centrifuged, and cell pellets and supernatant were collected and frozen in liquid nitrogen. 1 × 10^6^ sorted purified p190-BCR-ABL+ B-cell precursors from leukemic WT (*n =* 2) or *Klf5*^Δ/Δ^ (*n =* 3) mice, or from cultured WT + vector, WT + Gstm1, *Klf5*^Δ/Δ^ + vector and *Klf5*^Δ/Δ^ + Gstm1 groups were extracted in 1 ml of ice-cold lysis/extraction buffer (methanol: acetonitrile: water 5:3:2) as previously described [75]. Protein pellets were discarded and the water and methanol soluble fractions were run through a C18 reversed phase column (phase A: water, 0.1% formic acid; B: acetonitrile, 0.1% formic acid; Phenomenex, Torrance, CA) through an ultra-high performance chromatographic system (UHPLC - Ultimate 3000, Thermo Fisher). UHPLC was coupled in line with a high resolution quadrupole Orbitrap instrument run in either polarity modes (QExactive, Thermo Fisher) at 70,000 resolution (at 200 m/z). Maven software (Princeton), KEGG pathway database, and an in-house validated standard library (>650 compounds; Sigma Aldrich; IROA Technologies, Bolton, MA) were used for metabolite assignment and peak integration for relative quantitation. Integrated peak areas were exported into Excel (Microsoft, Redmond, CA) and elaborated for statistical analysis (*T*-test, ANOVA), principal component analysis (PCA – Multibase, Numerical Dynamics, Paris) and hierarchical clustering analysis (HCA) using GraphPad Prism (GraphPad Software Inc, La Jolla, CA) and GENE-E (Broad Institute) respectively.

### RNA-seq data analysis

Total RNA from sorted purified p190-BCR-ABL+B-cell precursors of leukemic WT (*n =* 3 samples) mice or *Klf5*^Δ/Δ^ (*n =* 3 samples) mice was isolated (RNeasy Micro Kit, Qiagen). RNA-seq was determined (Illumina HiSeq2500, Illumina, San Diego, CA) in the CCHMC DNA Sequencing and Genotyping Core following standard procedures. Data analysis was performed using the Strand NGS v3.0 software (Agilent Technologies). The differentially expressed genes in the *Klf5*^Δ/Δ^ versus WT samples were identified by applying the moderated *t*-test with the absolute fold change cut-off ≥2.0 and the *p*-value cut-off ≤ 0.05. Gene set over-representation analysis for the differentially expressed genes was performed by employing the Ingenuity Pathway Analysis v01–07 platform (IPA, Qiagen Bioinformatics) for the collections of GO gene sets and signaling pathways. The significance of gene set over-representation was estimated based on the hypergeometric test with the *p*-value cut-off ≤ 0.05. Differential expression was confirmed by real time (RT)-PCR for selected genes.

### shRNA screen analysis

The screen was performed from the Achilles v2.20.2 dataset (Broad Institute) [76] derived by integrating the next-generation sequencing deconvolution of 285 cell lines that passed quality control on an shRNA library of 98 k hairpins with the next-generation sequencing deconvolution of 216 cell lines that passed quality control on an shRNA library of 54 k hairpins. The Achilles v2.20.2 data were described by 501 cell lines from 31 cancer types on the combined libraries of 98 k and 54 k barcoded shRNAs in lentiviral vectors targeting 17,098 unique genes, out of which 11,234 genes have complete measurements for all 501 cell lines. The B-ALL lineage was represented in the Achilles v2.20.2 data by five out of the 501 cell lines: 697, NALM6, REH, RS411, SEM.

Dependency z-scores for each gene were identified based on the DEMETER computational method that uses the depletion values induced by each shRNA construct to infer the effect of suppressing its intended target (on-target) gene along with the effect of activating an off-target seed. DEMETER models each depletion value as a sum of two unobserved quantities: gene knockdown and seed-based effects and then estimates these quantities by fitting the model to the full dataset. The KFL5 dependency z-scores across the 501 lines were computed based on the three KLF5 hairpins with reliable scores for on-target gene suppression (TRCN0000280276, TRCN0000280277, TRCN0000280275). The differences in KLF5 dependency z-scores in B-ALL versus solid tumor cell lines in Achilles data were evaluated based on the Mann-Whitney nonparametric test with the cut-off 0.05 applied to the *p*-value. The genome-wide gene level dependency z-scores were illustrated through boxplots associated to individual lineages and through a hockey plot.

### Chromatin immunoprecipitation (ChIP) assay

Chromatin immunoprecipitation (ChIP) assay was performed (Magna ChIP™ A/G Chromatin Immunoprecipitation Kit, EMD Millipore, Catalog # 17-10085) according to the manufacturer’s protocol. Sorted purified WT p190-BCR-ABL+ B-cell precursors were prepared. Following the immunoprecipitation using anti-Klf5 antibody (Active Motif, Catalog # 61099), quantitative PCR (Q-PCR) was performed using the primers designed to encompass the putative Klf5 binding site. Immunoprecipitation using normal rabbit IgG and amplification of GAPDH gene serve as negative control.

### Statistical analysis

Data are presented as mean ± SD or mean ± SEM when several independent experiments have been analyzed. Statistical significance was determined using the unpaired Student-*t* test or Anova test, and differences in survival were examined using the Log-Rank test. Level of significance was established at *p* < 0.05.

## SUPPLEMENTARY MATERIALS FIGURES




