# Supplementary material for: KLF5 controls glutathione metabolism to suppress p190-BCR-ABL+ B-cell lymphoblastic leukemia
Source: Oncotarget. 2018 Jul 3;9(51):29665–79. doi: 10.18632/oncotarget.25667 (PMC6049869; doi:10.18632/oncotarget.25667)
Supplement: Supplementary file 1 [file oncotarget-09-29665-s001.pdf]

KLF5 controls glutathione metabolism to suppress p190-BCR-ABL+ B-cell lymphoblastic leukemia

SUPPLEMENTARY MATERIALS

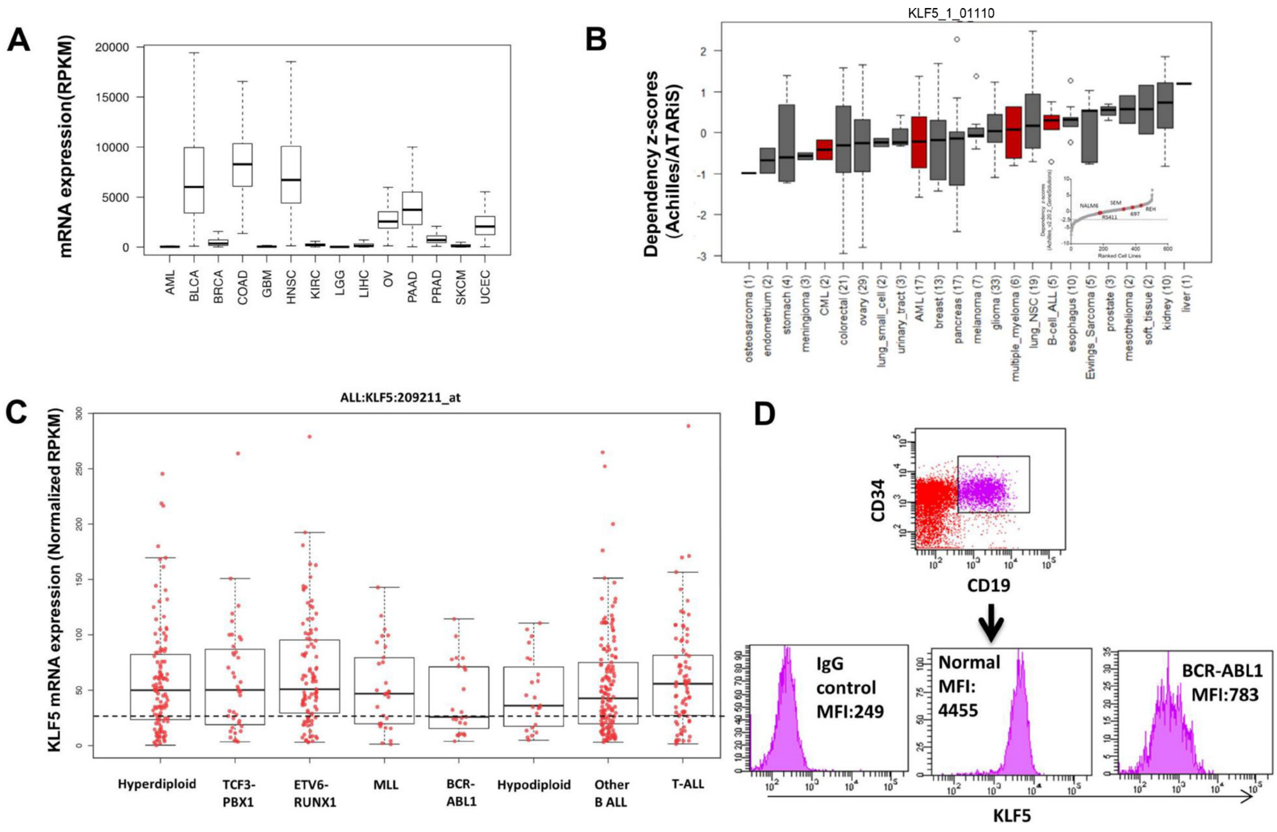

Supplementary Figure 1: KLF5 expression is differentially decreased in BCR-ABL1⁺ B-ALL. (**A**) KLF5 pan-cancer expression in The Cancer Genome Atlas (TCGA; https://cancergenome.nih.gov/) database (AML: acute myeloid leukemia; BLCA: Bladder Urothelial Carcinoma; BRCA: Breast invasive carcinoma; COAD: Colon adenocarcinoma; GBM: Glioblastoma multiform; HNSC: Head and Neck squamous cell carcinoma; KIRC: Kidney renal clear cell carcinoma; LGG: Brain Lower Grade Glioma; LIHC: Liver hepatocellular carcinoma; OV: Ovarian serous cystadenocarcinoma; PAAD: Pancreatic adenocarcinoma; PRAD: Prostate adenocarcinoma; SKCM: Skin Cutaneous Melanoma; UCEC: Uterine Corpus Endometrial Carcinoma). (**B**) Parallel genome-scale loss of function screens in 501 cancer cell lines from specific organs/tissues for the identification of context-specific genetic dependencies in the shRNA Achilles/ATARiS shRNA genomic library (KLF5 gene data analysis). Numbers between brackets denote the number of lines analyzed. Statistical analysis of B-ALL vs solid tumors: $P = 0.49$; for hematological vs solid tumors: $P = 0.76$; Mann–Whitney U test. Inset: Hockey plot with the non-BCR-ABL1 expressing B-ALL cell lines (n = 5) highlighted red. (**C**) KLF5 mRNA expression in pediatric B-ALL according to their genotypic classification as identified by the Pediatric Cancer Genome Project (https://www.stjude.org/research/pediatric-cancer-genome-project.html). (**D**) Representative KLF5 protein expression through flow cytometry graph of data shown in Figure 1C.

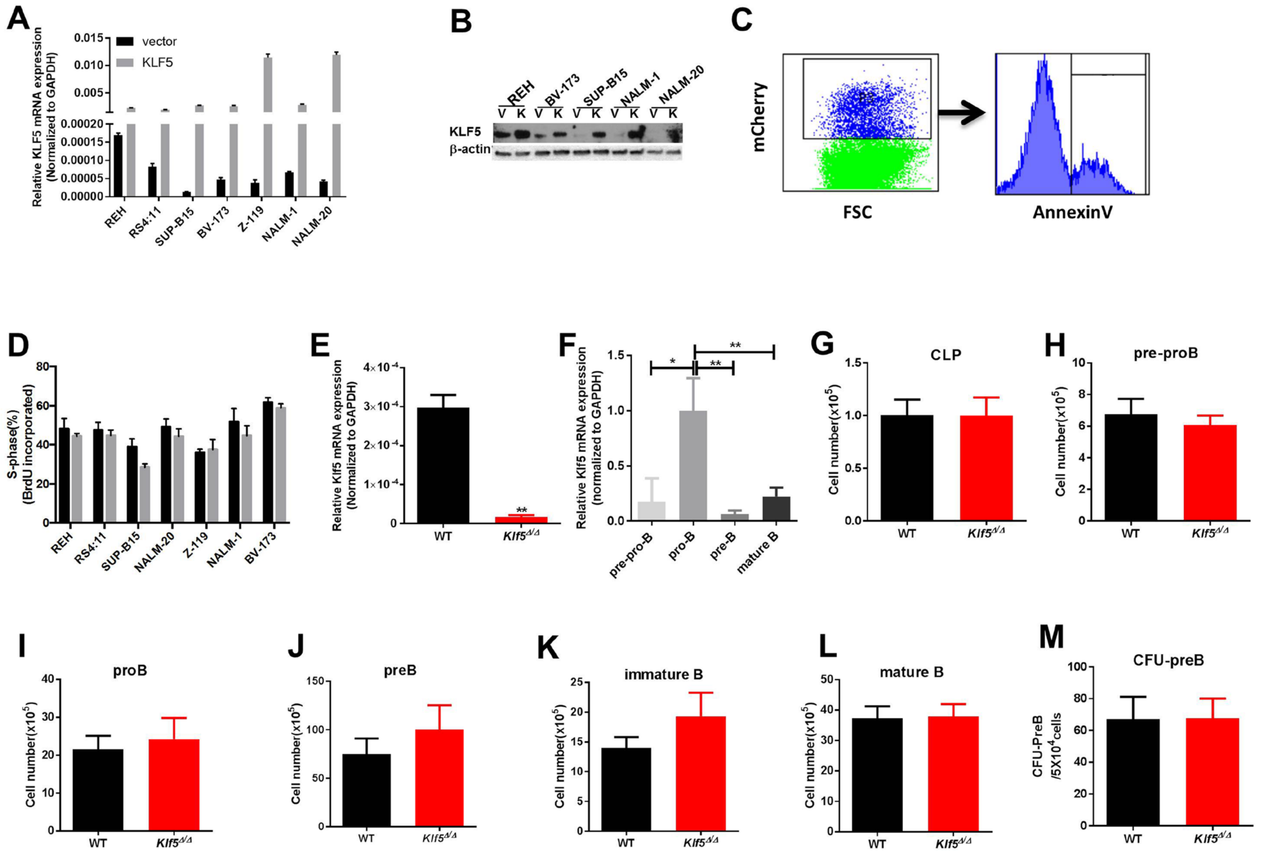

Supplementary Figure 2: Effect of Klf5 gain- and loss-of-function on leukemic and normal lymphoid precursors. (**A**) Relative mRNA expression of hKLF5 in human ALL cell lines transfected with empty vector or hKLF5 for each cell line. Values represented as mean ± SD. (**B**) Western blot showing hKLF5 expression in selected cell lines after transfection with empty vector or hKLF5. β-actin expression analysis from total lysate was used as a loading control. (**C**) Representative flow cytometry analysis gate set graph showed in Figure 1D, 1E. (**D**) Frequency of cells in S-phase as assessed by BrdU incorporation in B-ALL cell lines transduced with either KLF5 (grey bar) or empty vector (black bar). Data derived from two independent experiments. Each experiment was performed in duplicate and data are represented as mean ± SEM. (**E**) Klf5 mRNA expression in normal proB cells from *Vav1-Cre*; WT ($n = 3$) and *Vav1-Cre; Klf5* $^{flox/flox}$ ($n = 3$) mice. Values represented as mean ± SEM. (**F**) Klf5 mRNA expression in subpopulations of B cells from normal BM. Two independent experiments were performed in triplicates and data are represented as mean ± SEM. (**G**–**M**) Cell number of phenotypically identifiable B cell subpopulations (common lymphoid progenitors, CLP, (G); pre-proB cells (H); proB cells (I); preB cells (J); immature B cells (K); mature B cells (L) and CFU-preB (M)) in BM from *Vav1-Cre*; WT and *Vav1-Cre; Klf5*$^{flox/flox}$ mice. Data are presented as mean ± SEM from 6 mice per group.

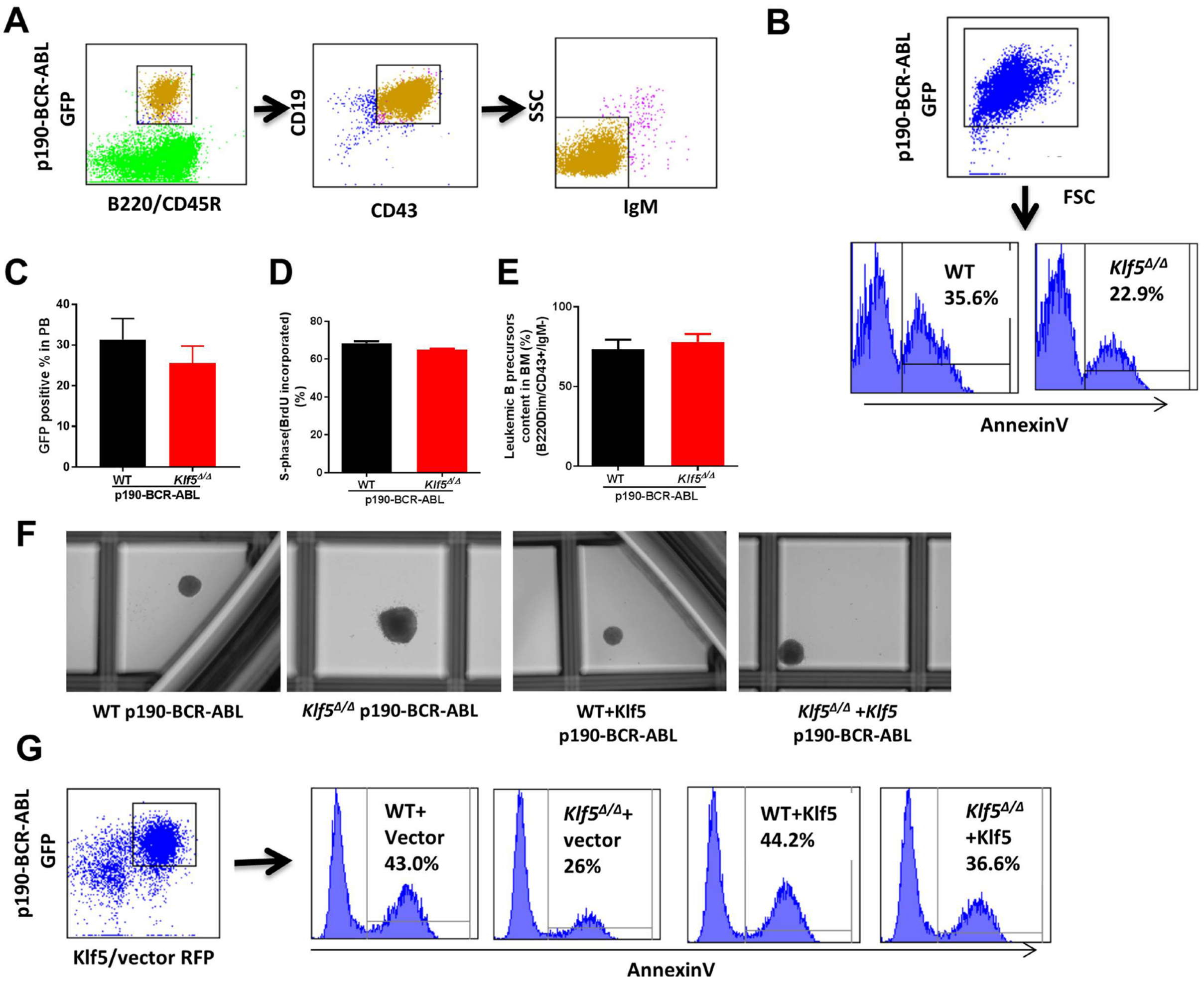

Supplementary Figure 3: Klf5 deficiency does not impair proliferation/differentiation of p190-BCR-ABL transduced B-cell precursors, just decreases their apoptosis. (**A**) Representative flow cytometry sorting gate set of early B-cell precursors either in vitro cultured cells or *in vivo* BM cells. (**B**) Representative flow cytometry graph about annexin V analysis showed in Figure 1H. (**C**) p190-BCR-ABL GFP+ percentage in Mx1-Cre; WT or Mx1-Cre; *Klf5$^{flox/flox}$* mice before administration of poly:IC. Data represented as mean ± SEM. (**D**) Frequency of cells in S-phase as assessed by BrdU incorporation in p190-BCR-ABL+ B-cell precursors from Mx1-Cre; WT (n = 3) or Mx1-Cre; *Klf5 $^{flox/flox}$* (n = 3) mice. Data represented as mean ± SEM. (**E**) p190-BCR-ABL+ B-cell precursors (B220 + dim/CD19+/CD43+/IgM−) content in BM from Mx1-Cre; WT (n = 16) or Mx1-Cre; *Klf5 $^{flox/flox}$* (n = 10) mice. Data are represented as mean ± SD. (**F**) Representative CFU-preB colony graph showed in Figure 2B. (**G**) Representative flow cytometry graph of annexin V analysis shown in Figure 2C.$^{*}P < 0.05$, $^{**}P < 0.01$.

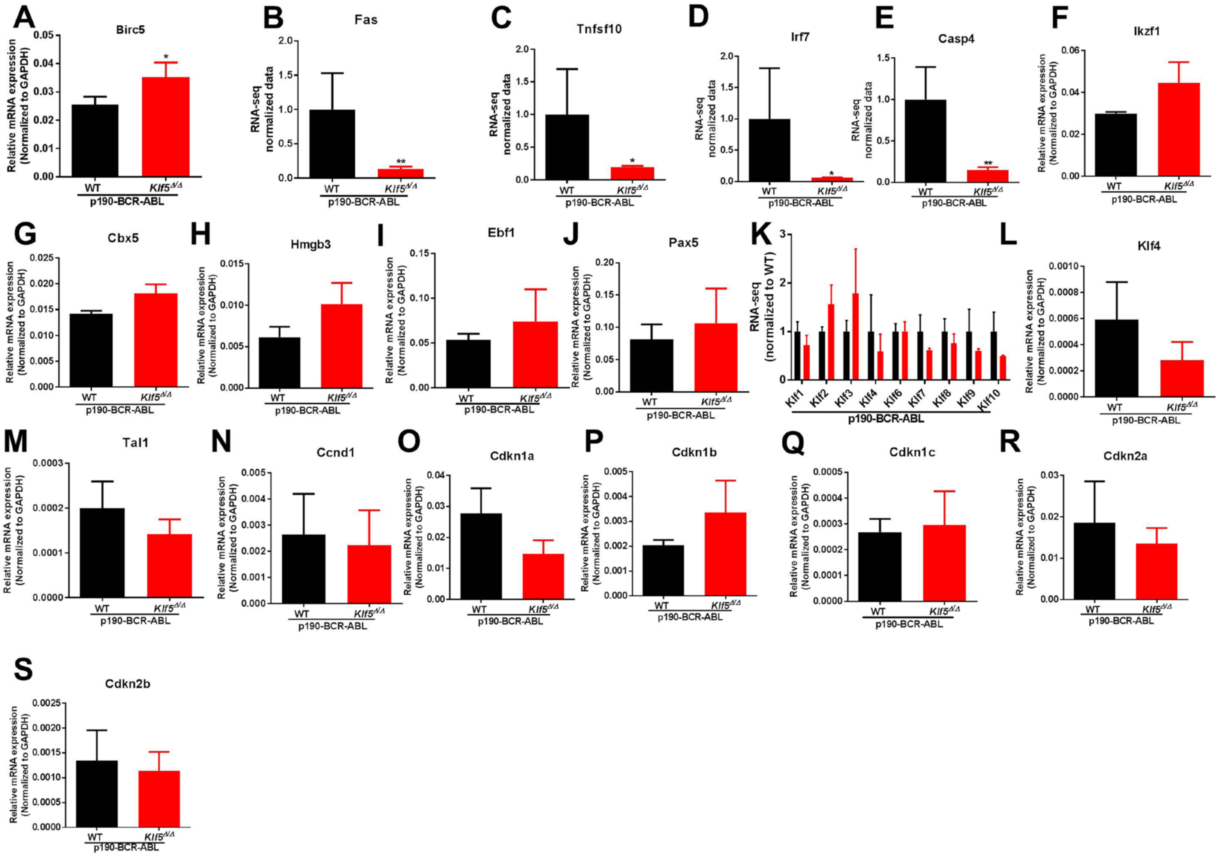

Supplementary Figure 4: Klf5 deficiency modifies apoptotic gene expression but does not modify the expression of cell cycle and differentiation related genes in p190-BCR-ABL transduced BM B-cell precursors. (**A**–**E**) Relative mRNA (Q-PCR) or RNA-seq normalized data showed expression of Birc5 (A), Fas (B), Tnfsf10 (C), Irf7 (D) and Casp4 (E) apoptosis related genes. (**F**–**J**) Relative mRNA expression of B cell differentiation related genes Ikzf1 (F), Cbx5 (G), Hmgb3 (H), Ebf1 (I) and Pax5 (J). (**K**–**L**) Other Klfs mRNA expression in p190-BCR-ABL transduced BM B-cell precursors (K RNA-seq normalized data, Black: WT; Red: *Klf5*$^{\Delta/\Delta}$), relative mRNA expression of Klf4 (L, Q-PCR). Relative mRNA expression of stem cell self- renewal genes Tal1 (M, Q-PCR). (**N**–**S**) Relative mRNA expression of cell cycle related genes Ccnd1, Cdkn1a, Cdkn1b, Cdkn1c, Cdkn2a and Cdkn2b derived from leukemic BM B-cell precursors from chimeric mice transplanted with *Mx1-Cre*; WT transduced with a mock vector (WT), *Mx1-Cre*; *Klf5*$^{flox/flox}$ transduced with a mock-vector (*Klf5*$^{\Delta/\Delta}$) or *Mx1-Cre*; *Klf5*$^{flox/flox}$ transduced with vector expressing mKLF5 (*Klf5*$^{\Delta/\Delta}$ + *Klf5)*. Data are represented as mean ± SEM from two independent experiments by triplicate. $^{*}P < 0.05$, $^{**}P < 0.01$.

Supplementary Data 1: Genes upregulated or downregulated >2 folds in *Klf5*$^{\Delta/\Delta}$ leukemic B-cell precursors compared with WT leukemic B-cell precursors (p < 0.05, n = 3 in each group). See Supplementary_Data_1
